# Long-Term Impact of Immunosuppressants at Therapeutic Doses on Male Reproductive System in Unilateral Nephrectomized Rats: A Comparative Study

**DOI:** 10.1155/2013/690382

**Published:** 2013-07-11

**Authors:** Yehui Chen, Zhi Zhang, Yun Lin, Huaxin Lin, Miaoyuan Li, Pin Nie, Lizhong Chen, Jiang Qiu, Yanmeng Lu, Linqiang Chen, Banglao Xu, Wuzhou Lin, Jing Zhang, Hong Du, Jianjian Liang, Zhiwei Zhang

**Affiliations:** ^1^Department of Urology, Guangzhou First People's Hospital, Guangzhou Medical University, 1 Panfu Road, Guangzhou, Guangdong 510180, China; ^2^Reproductive Center, Guangdong General Hospital, Guangdong Academy of Medical Science, Guangzhou, Guangdong 510080, China; ^3^Department of Organ Transplantation, The First Affiliated Hospital of Sun Yat-sen University, Guangzhou, Guangdong 510080, China; ^4^Laboratory of Electron Microscope, Southern Medical University, Guangzhou, Guangdong 510450, China; ^5^Department of Laboratory Medicine, Guangzhou First People's Hospital, Guangzhou Medical University, 1 Panfu Road, Guangzhou, Guangdong 510180, China; ^6^Department of Pathology, Guangzhou First People's Hospital, Guangzhou Medical University, 1 Panfu Road, Guangzhou, Guangdong 510180, China; ^7^Department of Pharmacy, Guangzhou First People's Hospital, Guangzhou Medical University, 1 Panfu Road, Guangzhou, Guangdong 510180, China; ^8^Division of Nephrology, Department of Medicine, VA Loma Linda Healthcare System and Loma Linda University, VA Loma Linda Healthcare System (111N), 11201 Benton Street, Loma Linda, CA 92357, USA

## Abstract

Cyclosporine, tacrolimus, and sirolimus are commonly used in renal transplant recipients to prevent rejection. However, information for comparative effects of these agents on the male productive system is extremely limited and controversial. In a physiologically and clinically relevant rat model of unilateral nephrectomy, we demonstrated that long-term oral administration of both cyclosporine and sirolimus at doses equivalent to the therapeutic levels used for postrenal transplant patients significantly affects testicular development and the hypothalamic-pituitary-gonadal axis accompanied by profound histological changes of testicular structures on both light and electron microscopic examinations. Spermatogenesis was also severely impaired as indicated by low total sperm counts along with reduction of sperm motility and increase in sperm abnormality after treatment with these agents, which may lead to male infertility. On the other hand, treatment with therapeutic dose of tacrolimus only induced mild reduction of sperm count without histological evidence of testicular injury. The current study clearly demonstrates that commonly used immunosuppressants have various impacts on male reproductive system even at therapeutic levels. Our data provide useful information for the assessment of male infertility in renal transplant recipients who wish to father children. Clinical trials to address these issues should be urged.

## 1. Introduction 

Renal transplantation has become the treatment of choice for patients with end-stage renal disease (ESRD) due to its superior survival benefit and quality of life. Although ESRD patients commonly experience sexual disturbance and reduced fertility, successful renal transplantation can restore these functions in both man and women [1–3]. Better understanding of the immune mechanisms for rejection of an allograft and the development of newer immunosuppressive drugs have also allowed more children who underwent renal transplantation to survive into adulthood, therefore prolonging the reproductive period in renal transplant recipients. 

 Cyclosporine, tacrolimus, and sirolimus are the most important components in commonly used immunosuppressive regimens, which have resulted in a dramatic improvement in the outcomes observed in renal transplant recipients over the past 30 years. Long-term administration of these medications, however, has contributed variably to the risks of cardiovascular disease, infection, malignancy, and nephrotoxicity, leading to the development of late mortality and chronic allograft dysfunction. Few animal studies also indicated that these drugs may be toxic to germ cells and impair both spermatogenesis and male gonadal function [4–6]. In humans, the sperm concentration and motility were inversely correlated with the cyclosporine whole blood trough levels [7] and gonadal dysfunction as well as infertility associated with sirolimus that have been reported in male renal transplant recipients [8–11]. 

 Previous translational studies that addressed the adverse effects of immunosuppressants are generally conducted in animals with two kidneys, which is not the case in renal transplant recipients who have only one functional kidney. The majority of studies were also relatively short term with the use of higher toxic doses of drugs via parental routes. Data on comparative effects of different agents are extremely limited. The present study was therefore designed to examine the impacts of long-term oral administration of commonly used immunosuppressants at therapeutic doses on testicular development and structural alteration, spermatogenesis, and gonadal function in male rats subjected to unilateral nephrectomy.

## 2. Materials and Methods

### 2.1. Materials

Male Sprague-Dawley rats (6-week old) weighting 160–180 g were provided by Guangdong Laboratory Animal Center. The animals were housed at constant temperature with a 12-hour light/dark cycle and allowed free access to standard rodent chow and tap water according to the policy established by the International Council for Laboratory Animal Science. All test substances are obtained from Hangzhou Zhongmei Huadong Pharmaceutical Co., Ltd (Hangzhou, China). 

### 2.2. Rat Model of Unilateral Nephrectomy

The animals were anesthetized by ether and surgically prepared and draped. A midline skin incision was made followed by blunt dissection to expose the left kidney. The kidney was then removed, and adequate hemostasis was ensured prior to closure of the abdomen with sutures. 

### 2.3. Experimental Design

Forty rats were randomly divided into 5 experiment groups of eight animals each: (A) control; (B) unilateral nephrectomy alone (UN); (C) UN plus cyclosporine; (D) UN plus tacrolimus; (E) UN plus sirolimus. After recovery from surgery, animals received daily treatment with either drinking water (Group A&B) or one of the drugs diluted in water (Group C to E) via oral gavage for 8 weeks. Since the first therapeutic doses of cyclosporin and tacrolimus in renal transplant recipients are 5 mg/kg/day and 0.15 mg/kg/day and the therapeutic dose for sirolimus is usually 2 mg/day, the doses of these agents to be administrated to the rats were 25 mg kg^−1^ day^−1^, 0.8 mg kg^−1^ day^−1^, and 0.2 mg kg^−1^ day^−1^, respectively, according to the formula *DW*_1_ = [(*R*_1_ *W*_2_^1/3^)/(*R*_2_ *W*_1_^1/3^)]*DW*_2_, where *DW* is drug dose, *R* is body index (0.09 for rat and 0.1 for human), and *W* is standard weight (200 g for rat and 60 kg for human). The concentrations of drugs in blood were measured to confirm their levels *in vivo* (data not shown). At the end of 8 weeks, blood was collected and bilateral testes were removed and weighed immediately. 

### 2.4. Sperm Analysis

Immediately after excision, the right testis was minced in 5 mL of normal saline solution pre-warmed to 37°C, incubated for 20 min at 37°C to allow the migration of all spermatozoa from testis tissue to fluid, and filtered via a double layer of lens filter papers to separate the supernatant from tissue particles. One milliliter of the sperm suspension was then mixed with trypan blue dye solution (1 mL to 0.1 mL) and examined in a hemocytometer chamber under light microscope. The total number of spermatozoa and the number of motile spermatozoa were counted, and the percentage of sperm mobility was calculated. A portion of the sperm suspension was also dropped on a glass slide, air dry, fixed with Bouin's solution, and examined under light microscope. A total of 200 spermatozoa were examined on each slide, and the percentage of sperm abnormality was calculated as sperm abnormality = number of head, tail, or total defect spermatozoa/200 spermatozoa examined.

### 2.5. Measurement of Serum Sex Hormone and Gonadotropins

The levels of sex hormone and gonadotropins were measured in serum samples by ELISA using commercially available assay kits according the manufacturer's instructions (R&D, USA). Briefly, 96-well microtiter plates were precoated with purified antibodies against individual rat sex hormone and gonadotropins. Fifty microliter of standard or samples (10 *μ*L of serum with 40 *μ*L dilutant) were added to each well and incubated for 30 min at 37°C, followed by washing for 5 times. Antibodies labeled with HRP (horseradish peroxidase) were then added to each well and incubated for 30 min at 37°C followed by washing for 5 times, and the reactions were developed by incubation with TMB (3,3′,5,5′-tetramethylbenzidine) substrate solution for 15 min at 37°C protected from light and terminated by the addition of a sulphuric acid solution. The color change was measured spectrophotometrically at a wavelength of 450 nm, and the concentrations of sex hormone and gonadotropins in each animal were then determined by comparing the OD (optical density) of the samples to the standard curve.

### 2.6. Histological Analysis of Testes

The left testis tissues were fixed in Bouin's fluid, embedded in paraffin, sectioned at 2 *μ*m, and stained with haematoxylin and eosin by routine techniques. The histological analysis was then performed using a laboratory upright microscope and the morphological changes of each specimen were examined by a pathologist who was blind to the sample subgroups. The degree of testicular injury was then scored according to the histological features as described in Table 1. For evaluation of ultrastructural changes of testes, tissue sections were fixed with 2.5% glutaraldehyde and 1% osmium tetroxide and embedded in epoxy resin. Ultra thin sections (60–90 nm) were stained with 2% uranyl acetate and 2% lead citrate prior to examination under a transmission electron microscope. 

### 2.7. Statistical Analysis

Statistical analysis was performed using a SPSS version 17.0 statistics program. Values were reported as means ± standard deviation. The one-way analysis of variance + post hoc LSD test and Mann-Whitney test were used as applicable. A *P* value of less than 0.05 was used to determine the level of statistical significance. 

## 3. Results

### 3.1. Body Weight and Testicular Development

**Figure 1:**
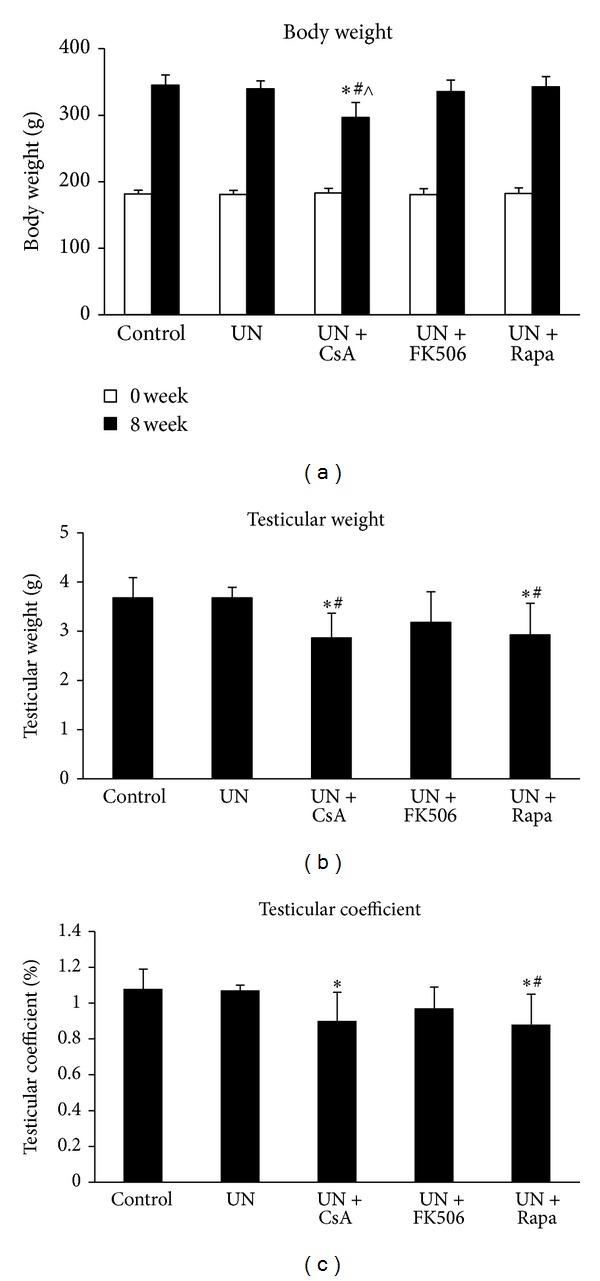
Body weight and testicular development in unilateral nephrectomized rats treated with cyclosporine, tacrolimus or sirolimus. Male Sprague-Dawley rats were randomly divided into 5 experiment groups of eight animals each. All animals except for the control group were subjected to left unilateral nephrectomy. After recovery from surgery, animals received daily oral gavage of either drinking water or one of the drugs diluted in water for 8 weeks and were weighed twice a week. The testes were harvested at the end of the study and weighed, and the testicular coefficient was calculated as the percentage of total body weight (testicular coefficient = bilateral testicular weight/total body weight). UN, unilateral nephrectomy; CsA, cyclosporine; FK506, tacrolimus; Rapa, sirolimus. *P < 0.05 compared with UN group; ^#^ *P* < 0.05 compared with UN+FK506 group; ^∧^ *P* < 0.05 compared with UN+Rapa group.

All animals survived to the end of 8-week, which is proportional to 5 human years [12]. As shown in Figure 1, the general health of these animals remained fair as indicated by continuous weight gain during the entire study period. Rats subjected to unilateral nephrectomy (UN) and given cyclosporine did not gain as much as the others. Normal testicular development was observed in UN rats and UN rats that received tacrolimus, but administration of cyclosporine or sirolimus in UN rats resulted in significant decline in testicular weight as well as testicular coefficient (Figures 1(b), and 1(c)), indicating a direct testicular toxicity associated with these agents.

### 3.2. Analysis of Spermatogenesis

**Figure 2:**
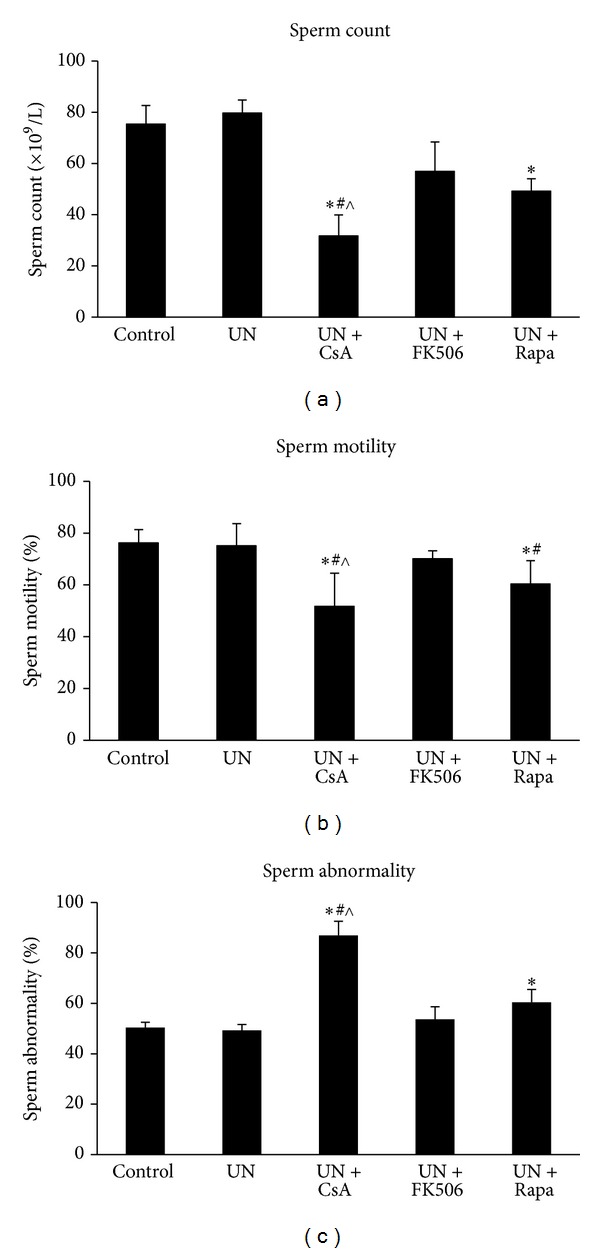
Analysis of spermatogenesis in unilateral nephrectomized rats treated with cyclosporine, tacrolimus, or sirolimus. Male Sprague-Dawley rats were randomly divided into 5 experiment groups of eight animals each. All animals except for the control group were subjected to tone left unilateral nephrectomy. After recovery from surgery, animals received daily oral gavage of either drinking water or one of the drugs diluted in water for 8 weeks. The testes were harvested at the end of the study, and sperm suspension was prepared from the testis sample for examination under light microscope. UN, unilateral nephrectomy; CsA, cyclosporine; FK506, tacrolimus; Rapa, sirolimus. *P < 0.05 compared with UN group; ^#^ *P* < 0.05 compared with UN+FK506 group; ^∧^ *P* < 0.05 compared with UN+Rapa group.

As shown in Figure 2, the characteristics of spermatogenesis were essentially the same in UN rats compared with controls. By contrast, total sperm count was significantly decreased in UN rats that received cyclosporine or sirolimus, although it was more profound with cyclosporine (Figure 2(a)). Reduction of sperm motility and increase in sperm abnormality were also remarkable in these animals (Figures 2(b) and 2(c)). On the other hand, treatment with tacrolimus in UN rats did not affect sperm motility or abnormality; although there was mild decrease in total sperm count, it was without statistical significance (Figure 2). These results indicated that administration of both cyclosporine and sirolimus but not tacrolimus induces profound impairment in spermatogenesis in UN rats.

### 3.3. Gonadal Function

**Figure 3:**
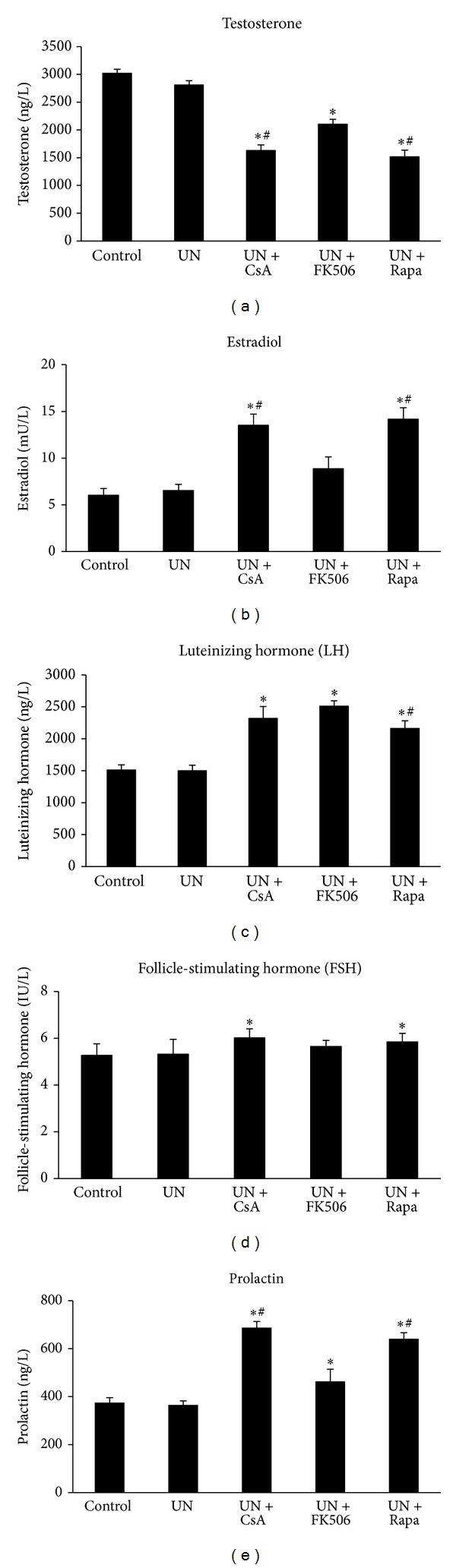
Levels of sex hormones and gonadotropins after treatment with cyclosporine, tacrolimus, or sirolimus in rats subjected to unilateral nephrectomy. Male Sprague-Dawley rats were randomly divided into 5 experiment groups of eight animals each. All animals except for the control group were subjected to left unilateral nephrectomy. After recovery from surgery, animals received daily oral gavage of either drinking water or one of the drugs diluted in water for 8 weeks. Blood was collected at the end of the study, and sex hormones (testosterone and estradiol) as well as gonadotropins (luteinizing hormone, follicle-stimulating hormone, and prolactin) were measured in serum samples by ELISA. UN, unilateral nephrectomy; CsA, cyclosporine; FK506, tacrolimus; Rapa, sirolimus. *P < 0.05 compared with UN group; ^#^ *P* < 0.05 compared with UN+FK506 group.

There were no differences in levels of sex hormone and gonadotropins between UN and control rats as shown in Figure 3. In contrast, treatment with cyclosporine or sirolimus in UN rats resulted in a significant reduction in testosterone level and elevation of estradiol level (Figures 3(a) and 3(b)). A less profound decrease in testosterone level without change of estradiol level was seen in UN rats which received tacrolimus (Figures 3(a) and 3(b)). On the other hand, levels of luteinizing hormone (LH), follicle-stimulating hormone (FSH), and prolactin were all significantly elevated in UN rats that received cyclosporine, sirolimus, or tacrolimus (Figures 3(c)–3(e)). These data demonstrated that administration of both cyclosporine and sirolimus has significant impact on the hypothalamic-pituitary-gonadal axis in UN rats, which in turn may impair male gonadal function and fertility. Treatment with tacrolimus affected the axis as well but not as profoundly as the others.

### 3.4. Histological Examination of Testicular Injury

**Figure 4:**
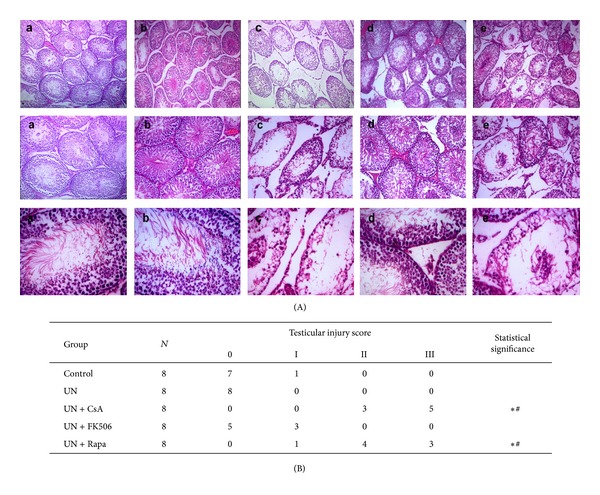
Histological examination of testicular injury in unilateral nephrectomized rats treated with cyclosporine, tacrolimus, or sirolimus. Male Sprague-Dawley rats were randomly divided into 5 experiment groups of eight animals each. All animals except for the control group were subjected to left unilateral nephrectomy. After recovery from surgery, animals received daily oral gavage of either drinking water or one of the drugs diluted in water for 8 weeks. The testes were harvested at the end of the study, and tissue sections were prepared for histological examination. (A) Morphological changes of seminiferous tubule architecture under light microscope in animals that received various treatments: a: control; b: unilateral nephrectomy (UN); c: UN plus cyclosporine; d: UN plus tacrolimus; e: UN plus sirolimus. Magnification, ×100 (upper panel); ×200 (middle panel); ×400 (lower panel). We examined 3 transversal sections of seminiferous tubules for each animal and one representative photo for each group was shown here. (B) Assessment of testicular injury by a histological scoring system as described in Table 1. Statistical significances between different treatments were determined by Mann-Whitney test using a SPSS program. CsA, cyclosporine; FK506, tacrolimus; Rapa, sirolimus. *P < 0.05 compared with UN group; ^#^ *P* < 0.05 compared with UN+FK506 group.

The histological changes of testicular structures in animals under various treatments are shown in Figure 4(A). Since male infertility is highly correlated with certain histopathological features of testes including loss of sperm and spermatids, disarray of germ cell layers, and absence of germ cell layers [13]; in the present study, we incorporated these histological parameters to develop a novel scoring system for the assessment of testicular injury more relevant to male infertility. Testicular injury was scored from 0 to III in each animal with the higher numbers indicating a more severe injury as described in Table 1. In both control and UN rats, light microscopic examination revealed a healthy appearance of the seminiferous tubule architecture with an orderly arrangement of germinal cells and plenty of sperm and spermatids (Figure 4(A)-a, b). In contrast, treatment with both cyclosporine and sirolimus in UN rats resulted in various degrees of atrophy of seminiferous tubes, loss of sperm and spermatids, disarray or absence of germ cell layers, decrease in spermatocytes, capillary congestion, and interstitial edema and fibrosis (Figure 4(A)-c, e). The testicular injury scores were significantly higher in these animals (Figure 4(B)). On the other hand, in UN rats that received tacrolimus, grossly normal seminiferous tubule architecture with only subtle decrease in sperm and spermatids and mildly increased testicular injury score without statistically significance was observed (Figures 4(A)-d and 4(B)). The ultrastructural changes of spermatocytes and Sertoli cells were also examined under transmission electron microscopy, which revealed various degrees of vacuole formation, mitochondria swelling, expansion of endoplasmic reticulum, and enlargement of the intramembranous space between nuclear membranes in UN rats that received both cyclosporine and sirolimus (data not shown).

## 4. Discussion

Hypothalamic-pituitary-gonadal dysfunction with decreased ovulation and sperm maturation is commonly seen in ESRD patients [14]. Gonadal dysfunction usually resolved by 6 months after successful renal transplantation followed by normalization of fertility [14]. Pregnancy is generally considered safe and feasible one year after renal transplantation as long as graft function is stable with stable immunosuppressant dosing at maintenance levels and without concurrent infections or use of teratogenic medications [14]. 

 Although a retrospective study with 185 patients in China showed that male recipients of renal transplant can get married and father children without significant effects on the children fathered afterwards and the function of allograft [1], few animal and human studies have indicated that certain immunosuppressants commonly used in renal transplant recipients might influence male gonadal and germ cell functions [4–6]. There are more than 60% of renal transplant recipients who are men and more than half of them are younger than 50, according to data from Organ Procurement and Transplantation Network (http://optn.transplant.hrsa.gov/). Concern about fertility in male renal transplant recipients at reproductive age on long-term use of immunosuppressive medications therefore needs to be addressed.

 Previous studies indicated that administration of cyclosporin results in a dose-dependent decline in body and reproductive organ weights, degenerative changes of seminiferous tubules, and impaired spermatogenesis in rats, which is not likely secondary to the potential hepatic or nephrotoxic effects of the drug [6, 15, 16]. Direct alteration in the hypothalamic-pituitary-gonadal axis [17–19], reduction in Sertoli cell phagocytic function [6], and oxidative stress in testicular tissues [20] have primarily contributed to the pathogenesis of the observed testicular and spermatozoal toxicities induced by cyclosporine. In contrast to cyclosporine, animals treated with subcutaneous injection of tacrolimus showed only dose-dependent decrease in sperm counts and motility without histopathological evidence of testicular injury [4] even though tacrolimus is 50–100 times more potent than cyclosporine [21]. Sperm counts and motility can return to control levels after stopping the drug [4]. However, it was also reported that significant histopathological changes in the seminiferous tubules along with spermatogenic damage and reduction in the number of Sertoli cells could be seen in rats after prolong subcutaneous administration of tacrolimus (30 to 60 days) [22]. Sirolimus, on the other hand, is known to block spermatogenesis by interrupting the crucial stem cell factor/c-kit system via inhibition of a rapamycin-sensitive PI3K/Akt/P70S6K/cyclinD3 pathway [23, 24]. It is therefore not surprising that azoospermia or oligozoospermia as well as infertility has been reported in male renal transplant recipients who received sirolimus [9–11]. Significant reduction of testicular weight and alterations of sexual hormone production, seminiferous tubule morphology, and spermatogenesis were also observed in animals treated with intraperitoneal injection of sirolimus, although withdrawal of this drug could lead to complete recovery of these effects [5]. 

 The present comparative study was designed to evaluate the impacts of commonly used immunosuppressants on male reproductive system in a physiologically and clinically relevant manner. The doses of drugs applied to experimental animals were proportional to the therapeutic doses used for postrenal transplant patients and were administrated orally for a long period of time (8 weeks). Since renal transplant recipients have only one functional kidney, all animals treated with these drugs were subjected to unilateral nephrectomy (UN). Similar to previous findings, our study revealed a reduction of body weight gain and testicular weight, a decrease in serum testosterone level along with elevated levels of gonadotropins, significant impairment of spermatogenesis, and evidence of severe testicular injury in both light and electron microscopic examination in UN rats that received cyclosporine. By contrast, administration of tacrolimus in UN rats induced only mild but statistically insignificant changes of spermatogenesis without effects on body weight gain and testicular development or evidence of testicular injury, although the testosterone level was reduced along with elevated LH level. Our results indicated that the mild impairment of spermatogenesis observed in tacrolimus-treated UN rats is likely secondary to its effect on the hypothalamic-pituitary-gonadal axis rather than a direct injury to the testes, which is not in agreement with data from another study using a higher toxic dose via parental administration of the drug in rats without nephrectomy [22]. The recent findings describing profound testicular toxicity associated with sirolimus [5] were confirmed in the current study. In UN rats treated with sirolimus, we observed significant histological changes of testicular structure in both light and electron microscopic examinations along with severe impairment of testicular development and spermatogenesis as well as male gonadal dysfunction. The overall abnormalities associated with administration of sirolimus are similar to cyclosporine but much more severe than tacrolimus. Fortunately, the sirolimus-associated testicular toxicity and infertility are potentially reversible [5], and significant improvement in spermatogenesis and restoration of fertility have been reported in male renal transplant recipients after changing sirolimus to tacrolimus [9–11]. 

 Although a large number of renal transplant recipients have been routinely treated with cyclosporine or sirolimus-based immunosuppressive regimens, clinical information concerning the impact of long-term administration of these drugs on sexual function and male fertility is very limited. Certainly there is urgent need for randomized-control trials to address these issues. Until such data become available, both transplant physicians and transplant recipients should be aware and informed of the potential adverse effect of cyclosporine and sirolimus on male reproductive system. Patients receiving either cyclosporine or sirolimus-based immunosuppressive regimens who wish to father children may need to be routinely monitored for their fertility status. Since these adverse effects are potential reversible especially for sirolimus, patients who attempt to father a child but are unsuccessful should be evaluated for substitution by tacrolimus or other immunosuppressants. 

 In summary, our comparative study demonstrates that long-term oral administration of commonly used immunosuppressants at therapeutic doses has various effects on male reproductive system in rats subjected to unilateral nephrectomy, which is physiologically and clinically relevant to postrenal transplant state in humans. Both cyclosporine and sirolimus caused significant testicular injury and profound alterations on the hypothalamic-pituitary-gonadal axis, resulting in severe impairment of spermatogenesis and likely male infertility. Tacrolimus, on the other hand, induced only mild changes of spermatogenesis without histological evidence of testicular injury. Our finding provides important information for evaluation of male infertility and selection of immunosuppressive regimens in renal transplant recipients who wish to father children. 

## 5. Conclusions

The current study clearly demonstrates that commonly used immunosuppressants have various impacts on male reproductive system even at therapeutic levels. Our data provide useful information for assessment of male infertility in renal transplant recipients who wish to father children. Clinical trials to address these issues should be urged.

## Figures and Tables

**Table 1:** Histological scoring system for assessment of testicular injury.

Score	Histological features of seminiferous tubules	Remark
0	Orderly arrangement of germinal cells with plenty of sperm and spermatids	Normal
I	Preservation of germ cell layers with decrease of sperm and spermatids	Mild injury
II	Disarray and decrease of germ cell layers with loss of sperm and spermatids	Moderate injury
III	Absence of germ cell layers	Severe injury
